# Internal Spreading of Papillary Thyroid Carcinoma: A Case Report and Systemic Review

**DOI:** 10.1155/2018/7618456

**Published:** 2018-01-08

**Authors:** Hui Jin, Huanhuan Yan, Huamei Tang, Miao Zheng, Chaojie Wu, Jun Liu

**Affiliations:** ^1^Shanghai General Hospital of Nanjing Medical University, Shanghai 201620, China; ^2^Department of Breast-Thyroid-Vascular Surgery, Shanghai General Hospital, Shanghai Jiaotong University, Shanghai 201620, China; ^3^Pathological Center of School of Medicine, Shanghai Jiaotong University, Shanghai 201620, China

## Abstract

An 18-year-old female diagnosed finally as PTC with intrathyroid spread was reported, and the diagnosis and surgical treatment of internal spreading of PTC were discussed. One lump was found on the thyroid isthmus by physical examination and B ultrasound, and multiple nodular shadows were found by CT. This patient finally underwent total thyroidectomy with bilateral central node dissection due to multifocal papillary thyroid carcinoma except PTC in the isthmus found in right lobe by intraoperative frozen section. The pathological section showed a major thyroid carcinoma in thyroid isthmus with scattered micropapillary carcinoma around it in the whole thyroid gland. The small lesions are distributed around central lesion in a radial form and the number of small lesions decreases with increased distance from central lesion. PTC with internal spread should be distinguished from multifocal PTC and poorly differentiated PTC in pathology. Thyroid cancerous node had a large diameter; it was likely to have internal spread. Combined imaging before surgery should be valued to diagnose PTC with internal spread. Preoperative CT and intraoperative frozen section are helpful for surgical volume selection of PTC with internal spread.

## 1. Case Abstract

An 18-year-old female patient was hospitalized due to “a touchable lump on the right of thyroid isthmus” which has been found for about 2 months. The patient has congenital heart disease history and no thyroid disease history in her family.* Physical Examination.* Bilateral thyroid was slightly swollen. A hard lump about 20 × 10 mm could be touched on thyroid isthmus and could move up and down when swallowing. No vascular murmur could be heard.* Laboratory Examination*. Routine blood examination and the level of thyroid hormone were normal.* B Ultrasound*. Echo of thyroid was even, and a low echo tubercle, with size 11 × 20 mm, aspect ratio 0.55, unclear border, and irregular shapes, could be observed on the right side of thyroid isthmus. Multiple dot-like calcification and blood flow signals could be observed (Figure 1(a)).* CT Scan*. Multiple nodular shadows of lower density could be observed in thyroid and some were calcified with a larger one located in isthmus, about 17 × 10 mm, and the rest were normal (Figure 1(b)).* FNA*. Cells were arranged closely and overlapped presenting papillary structure. Cell nucleus ditches could be observed (Figure 1(c)). We diagnosed it preoperatively as papillary thyroid carcinoma (PTC).* Echocardiography*. Congenital heart disease with ventricular septal defect, left to right shunt, and mild pulmonary hypertension.

A hard lump of size 20 × 15 mm and unclear border could be seen on the isthmus of thyroid during surgery. Nanometer carbon tracer was injected into bilateral thyroid to help us to distinguish lymph nodes from parathyroid glands. At first, the right thyroidectomy + isthmectomy with right central node dissection was performed. However, the intraoperative frozen section not only confirmed the preoperative diagnosis of PTC on the thyroid isthmus, in which disordered and branched papillary structure, scattered psammoma bodies, frosted-glass-like cell nucleus, larger nucleus, nucleus grooves, and intranuclear pseudoinclusions were found (Figure 2(b)), but also showed multifocal papillary thyroid carcinoma (MPTC) in right thyroid lobe as multiple psammoma bodies and papillary structure could be found in it (Figure 2(a)). Subsequently, left thyroidectomy and left central node dissection were added, and then the operation was finally ended. Multiple psammoma bodies and heterotypic cells were also found in left thyroid lobe on intraoperative frozen section (Figure 2(c)). The patient was discharged 3 days after operation without any symptoms of hypoparathyroidism and recurrence of laryngeal nerve injury.

Postoperative pathology showed that micropapillary carcinomas scattered around a major thyroid carcinoma (22 × 11 × 20 mm) in thyroid isthmus, and scattered micropapillary carcinoma also existed on both thyroid right lobe and left lobe (Figures 3–5). Immunohistochemistry showed CK19 (+), HBME-1 (+), TG (+), TTF-1 (+), TPO (+), CD56 (−), ki67 (about 3%), and beta-catenin (+). And 2 out of 3 central lymph nodes with metastasis were found. Interestingly, it can be observed that some small lesions are distributed around central lesion in radial form and the number of small lesions decreases with increased distance from central lesion on postoperative pathological sections. A larger carcinoma lesion (lower and right corner) with some small lesions composed of tens of heterocysts (upper-left section) can be observed in Figure 3. A spread lesion in the lymph-vessel blackened by nanometer carbon tracer and blackened lymph-vessels in the lesion were showed in Figure 4. Intraoperative frozen sections were analyzed again and the number of satellite lesions in the left lobe and right lobe was counted under the same magnification. It can also be observed that some small lesions are distributed around central lesion in radial form and the number of satellite lesions decreases with increased distance from central lesion, and the central lesion is just the PTC in the isthmus near the right lobe. The number of satellite lesions in the right lobe is more than that in left lobe (Figure 5). Therefore, the final pathological diagnosis was considered as PTC with intrathyroid spread.

## 2. Discussion

### 2.1. Diagnosis and Identification of Internal Spread of PTC

PTC is the most common type of thyroid cancer, taking around 70%* *~80% [1]. Internal spread of PTC is one of the metastasis ways of thyroid cancer. There is a connection among lesions including a major one and small lesions in the surrounding parts. It is usually spread by the abundant lymphatic networks in thyroid. In this case, it can be observed from postoperative pathological sections that some small lesions are distributed around central lesion in radial form and the number of small lesions decreases with increased distance from central lesion. Therefore, it was considered as internal spread of thyroid carcinoma. A larger carcinoma lesion has increased possibility of internal spread. When a satellite lesion is as small as the accumulation of tens of cancer cells, medical imaging does not always reflect abnormal signs; when it grows to a certain size, preoperative diagnosis is similar to MPTC.

PTC with internal spread should be distinguished from MPTC and poorly differentiated papillary carcinoma. MPTC is one of the clinical characteristics of PTC and exists as multiple independent carcinoma lesions in thyroid. Its malignancy is higher than single lesion carcinoma of thyroid. It is closely associated with lymphatic metastasis, recurrence, and prognosis. The incidence of MPTC is about 18%* *~87% in PTC [2, 3]. MPTC is one form of PTC and featured by invasion into lymph node and tissue around thyroid and recurrence. The lymphatic metastasis rate of MPTC is close to 60%. MPTC can be classified into three types according to the locations: (1) MPTC in unilateral thyroid: preoperative diagnosis finds no tubercle in opposite thyroid; (2) MPTC in unilateral thyroid: preoperative diagnosis finds tubercles in opposite thyroid but considers them as benign tubercles; (3) MPTC are distributed in bilateral thyroid. Hashimoto's thyroiditis (HT) and hereditary thyroid cancer are the risk factors of MPTC. Other clinical statistics also reflect PTC combined with HT has higher incidence of MPTC [4, 5]. HT affects the whole thyroid gland. It finally results as diffuse destroyed thyroid cells and higher TSH level, and the latter may stimulate the proliferation of papillary cancer cells. The malignant tubercle developed based on this type of diffuse lesion often bears multiple lesions [6, 7]. The tubercles of multilesion carcinoma are similar and not directly associated. According to the available clinical data, internal spread of PTC is a special type for PTC. Compared with MPTC, the surrounding lesions are smaller and only can be observed under a microscope. As it is not a macroscopic tubercle, it cannot be touched in surgery. Under microscope, larger cancerous tubercles surrounded by tens or hundreds of small cancerous nests in radial form can be observed. The number of small tubercles and cluster degree decrease with increased distance from large tubercles, which indicates internal spread of thyroid carcinoma.

Poorly differentiated thyroid cancer is seldom composed of single structure and often exists as the combination of island, beam, or solid structures in different proportions. Solid structure is very common. The tumor cells do not have the thyroid follicles typical nuclear features of PTC, spread along the lymphatic vessels and thyroid follicles side clearance.

### 2.2. Preoperative Diagnosis of Internal Spread of Thyroid Papillary Carcinoma

**Figure 1:**
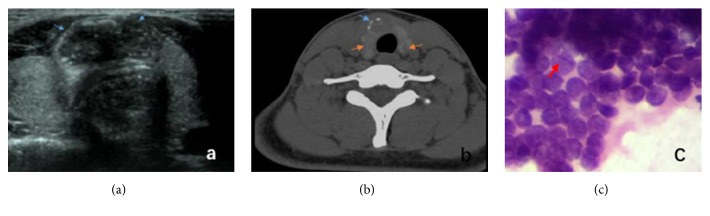
(a)* B Ultrasound*. Blue arrow for the low echo tubercle in the thyroid isthmus. (b)* CT Scan*. Yellow arrow for thyroid multiple lower density nodule; blue arrow for calcification. (c)* FNA*. Red arrow for cell nucleus ditch.

While it is easy to make preoperative diagnosis of PTC, it is extremely difficult to diagnose internal spread of thyroid papillary carcinoma. B ultrasound is a preferred medical imaging means to diagnose a thyroid disease. If a tubercle of low echo, irregular form, unclear border, and calcification is found, the possibility of thyroid cancer should be considered. According to historical clinical data analysis, if a tubercle is found to have any 3 features above, over 90% of pathology literature reports the specificity of malignant tubercle. In this case, the low echo tubercle in isthmus has irregular form, unclear border, and strong echo, which highly suggests the possibility of malignant tubercle. For suspected malignant tubercle and the lymph gland with possibility of metastasis, fine needle aspiration (FNA) under guidance of B ultrasound is very significant to the identification of thyroid tubercle, malignant or not, and determination of lymph node dissection scope. As proved by multiple statistics, the coincidence rate of FNA diagnosis and postoperative pathological sections is above 90% [8]. According to the result of FNA for calcified tubercles, it was found that cells are arranged closely and overlapped presenting papillary structure. Nucleus grooves of cells could be seen. The case was diagnosed as PTC (Figure 1(c)).

It can be found from this case that CT is very significant to comprehensive diagnosis of thyroid diseases. Due to the damaged iodine-storing cells in thyroid cancer, a low-density zone can be seen in CT image. In this case, CT image showed multiple low-density tubercle shadows in addition to the tubercle in isthmus, and B ultrasound image showed even echo of thyroid. We tried to analyze that, in the early stage of internal spread of thyroid papillary carcinoma, a macroscopic tubercle was not yet formed in the surrounding but the gather of cells only. US is the common method for thyroid cancer diagnosis with high sensitivity within 1 mm. CT is a useful complement for US in judging the invasion of adjacent tissues, and the involved lymph node in some areas [9–11]. Why were the multiple low-density tubercle shadows found by CT but not by US in our case? One reason may be that US evaluation is operator dependent. The other reason may be that US is not sensitive to this kind of small foci of intrathyroid spread of PTC.

For the diagnosis of internal spread of PTC, it is essential to combine multiple diagnosis means based on medical history and correctly judge the distribution of major tubercle and surrounding lesions and metastasis of neck lymphatic. In this case, B ultrasound report hinted a tubercle is at the right side of isthmus while CT showed multiple low-density tubercle shadows. As B ultrasound was limited by human factors, diagnosis scope might be shrunken resulting in a negative result, and the CT scanning way could just make up this defect of B ultrasound.

### 2.3. Intraoperative Frozen Pathology Section Is Helpful to Guide the Way of Surgery in the Case of Internal Spread of PTC

In this case, according to the result of B ultrasound and CT before surgery, the tubercle was located on the right side of isthmus. Meanwhile, considering the age of the patient, the primary plan was to resect right thyroid and its isthmus and perform central lymph node dissection. Result of intraoperative frozen pathology section showed that micropapillary carcinomas were dispersed in the isthmus of thyroid gland and right thyroid. After knowing this result, we considered the possibility of MPTC, and resect the left lobe, and sent for intraoperative frozen pathology. The result showed psammoma bodies and some heterocyst. We considered it as micropapillary carcinomas. If according to preoperative imaging and NCCN guideline only, resection scope included right lobe, isthmus and central lymph node. The result of intraoperative frozen pathology section guided us to resect all thyroid tissue and central lymph node. We thought that the satellite lesions were small in the early stage of metastasis, not large enough to be found in imaging. If internal spread of thyroid papillary carcinoma was not certain, we should pay attention to the result of intraoperative frozen pathology section.

If the case was diagnosed as internal spread of PTC, surgery scope should refer to MPTC. For internal spread of thyroid, resection scope does not vary greatly with MPTC. Most statistical results support whole resection, even though central lesion and satellite lesion are limited to one side. Related data statistics about internal metastasis of PTC are still in short supply. There are research reports about primary multifocal carcinoma. For the cases diagnosed as single-sided multifocal PTC, offside thyroid was cut within half a year and the relevance ratio of opposite neoplastic foci was as high as 69.1%. For patients with HT of B ultrasound and thyroid function, even though multiple lesions are in single side, both sides should be cut off. Multifocal carcinoma is an independent risk factor of lymphatic metastasis. It is safe to perform whole cutting and central lymph node dissection. For skipping lymphatic metastasis, functional lymph node dissection should be performed.

## 3. Conclusion

This is a typical case of internal spread of PTC as a major thyroid carcinoma in thyroid isthmus with scattered micropapillary carcinoma around it in the whole thyroid gland. PTC with internal spread should be distinguished from multifocal PTC and poorly differentiated PTC in pathology. Combined imaging, as B ultrasound, CT, and FNA, before surgery should be valued. Sometimes, even though B ultrasound report showed no abnormality of multiple lesions, we could not eliminate the possibility of internal metastasis of PTC. Especially when cancerous nodule had a large diameter, it was likely to have internal metastasis. Preoperative CT and intraoperative frozen section are helpful for surgical volume selection of PTC with internal spread.

## Figures and Tables

**Figure 2:**
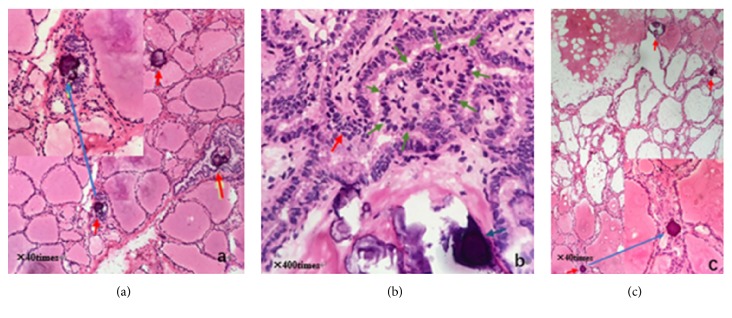
(a) Intraoperative frozen pathology of right thyroid lobe: red arrow for psammoma body. (b) Intraoperative frozen pathology of thyroid isthmus: red arrow for heterocyst; blue arrow for psammoma body; green arrow for papillary structure. (c) Intraoperative frozen pathology of left thyroid lobe: red arrow for psammoma body.

**Figure 3:**
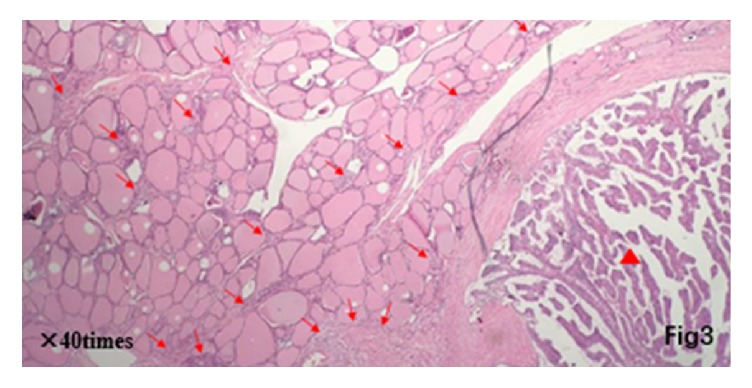
Red triangle for large carcinoma lesions at the lower right corner; red arrow for small lesions composed of dozens of heterocyst reunion on the left side.

**Figure 4:**
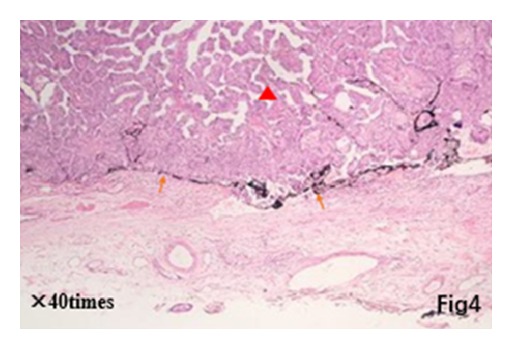
Red triangle for large carcinoma lesions; yellow arrow for tracer dye black lymphatic vessels.

**Figure 5:**
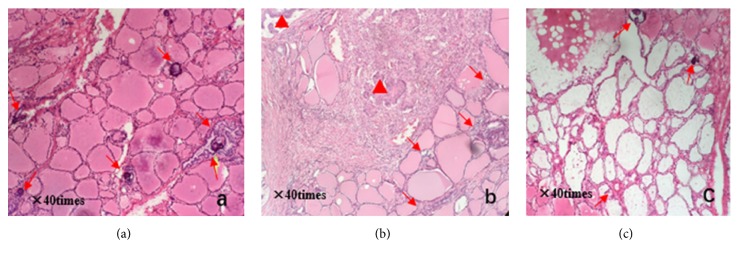
(a) Pathology section of right thyroid lobe: red arrow for psammoma body. (b) Pathology section of thyroid isthmus: red triangle for large carcinoma lesions; red arrow for small lesions composed of dozens of heterocyst. (c) Pathology section of left thyroid lobe: red arrow for psammoma body.
